# Access to urban green spaces in Hannover: An exploration considering age groups, recreational nature qualities and potential demand

**DOI:** 10.1007/s13280-022-01808-x

**Published:** 2022-12-12

**Authors:** Raphael Weber, Annegret Haase, Christian Albert

**Affiliations:** 1 Institute for Geography, Leipzig University, Johannisallee 19a, 04103 Leipzig, Germany; 2 Department of Urban and Environmental Sociology, Helmholtz Centre for Environmental Research – UFZ, Permoserstr. 15, 04318 Leipzig, Germany; 3 Institute of Geography, Environmental Analysis and Planning in Metropolitan Regions, Ruhr-University Bochum, Universitätsstr. 150, 44801 Bochum, Germany

**Keywords:** Accessibility, Age groups, Geographic information system analysis, Landscape planning, Recreation, Urban green space

## Abstract

City dwellers' accessibility of urban green spaces (UGS) has recently gained immense interest in research and policy. Related scientific studies thus far have focused primarily on spatial distances, largely missing considerations of UGS qualities. We analysed the entire UGS setting of Hannover considering the recreational nature quality and potential demands to identify age-appropriate green spaces by applying a geographic information system analysis of several data sets. Additionally, we assessed the accessibility of UGS for different age groups, varying recreational nature qualities, and potential demands. Results indicate that children and elderly people have poor access to UGS that offers age-related requirements to enable unrestricted nature-based recreation. Nature quality and age-related requirements play a significant role in the assessment of UGS for recreation and accessibility. We conclude that detected vulnerabilities regarding age-related recreation in cities are anchors to mainstream the issue and enhance future planning practices and research.

**Supplementary Information:**

The online version contains supplementary material available at 10.1007/s13280-022-01808-x.

## Introduction

As part of the Sustainable Development Goals, governments have pledged to “make cities and human settlements inclusive, safe, resilient and sustainable” by 2030. Urban green spaces (UGS) can play a major role in enhancing the quality of life in cities by safeguarding, enhancing, and restoring ecosystem services (ES). Many studies have pointed out the positive effects of UGS on human health (Konijnendijk et al. 2013; Markevych et al. 2014), with recreational ES as a key contributor to cultural ES (Hermes et al. 2018).

However, urban dwellers can only obtain these recreational benefits if they are next to or in the UGS. Therefore, a residential proximity of walking distance to the UGS for daily recreation is important to ensure healthy environments in cities. Along this line, the World Health Organization (WHO 2010, p. 6) declared “to provide each child by 2020 with access to healthy and safe environments and settings of daily life in which they can walk and cycle to […] green spaces in which to play and undertake physical activity”. In Germany, several municipalities^1^ and the council for landscape conservation proposed standards on near-residential open spaces to provide UGS access (Deutscher Rat für Landespflege [DLR] 2006).

Environmental equity has recently evolved into a mainstream lens through which scholars investigate accessibility issues in environmental policy and planning debates. The primary challenge is to enhance the access to and state of environmental goods for urban residents in an equitable and fair manner. Recent studies have analysed the distribution of UGS in relation to socio-demographic and user data, or other characteristics attributed to population groups and their social status (Comber et al. 2008; Kabisch and Haase 2014; Raymond et al. 2016). However, there remains a lack of integrated research that qualifies recreational green spaces, access, and user groups in an equity-oriented fashion.

Especially in Germany, studies dealing with the provision of and access to UGS often focus on large-scale spaces such as cities, regions, or even the entire country (Krekel et al. 2016; Grunewald et al. 2017; Richter et al. 2017; Wüstemann et al. 2017). Only a few studies have investigated the accessibility and recreational value of UGS at the small-scale and neighbourhood levels (ibid.; Kabisch and Haase 2014).

This paper aims to explore the relationship between the accessibility of UGS, their recreational nature quality (RNQ), and the socio-demographic characteristics of nearby residents in a case study based on Hannover city in Germany. Our research questions include: (i) What is the RNQ of UGS in Hannover? (ii) To what extent are suitable UGS available for different age groups? (iii) How does the accessibility of UGS relate to the different age groups of city-dwellers, also considering the varying levels of RNQ and Potential Demand of UGS?

## Conceptual framing

This study uses the environmental justice lens as a frame and motivation to explore the equitable accessibility of UGS among different groups of dwellers. Environmental Justice is based on the perspective “to achieve equitable protection from environmental harms and facilitate access benefits [for] everyone” (Ravi et al. 2021, p. 6). We use the distributional and interactional dimensions according to Kabisch and Haase (2014). We thus understand Environmental Justice in an equity-oriented manner, highlighting the Potential Demands of social groups for improved and just recreation spaces.

The focus of contemporary research literature on UGS and environmental justice moved from only quantitative considerations (“the more the merrier”, see van den Bosch et al. 2015, p. 7984) to a stronger consideration of qualitative aspects. Varying qualities of nature receive more emphasis, especially when considering just liveability and health aspects through recreation. Research on age-related recreational preferences has also received increasing attention in urban landscape planning, especially for elderly people (Hermes et al. 2018; Wen et al. 2018). In this study, we employ the concept of RNQ, which builds on perceived sensory dimensions that were developed by questionnaires based on nine towns and cities close to Stockholm, Gothenburg, and Malmö (Grahn and Stigsdotter 2010). Additionally, the RNQ approach was further qualified by integrating field trips, inventories, and more than 50 interviews conducted in UGS (ibid.; Berggren-Bärring and Grahn 1995; Grahn et al. 2005; Annerstedt et al. 2012; van den Bosch et al. 2015; Gao et al. 2019).

## Materials and methods

### Hannover city case study

**Fig. 1:**
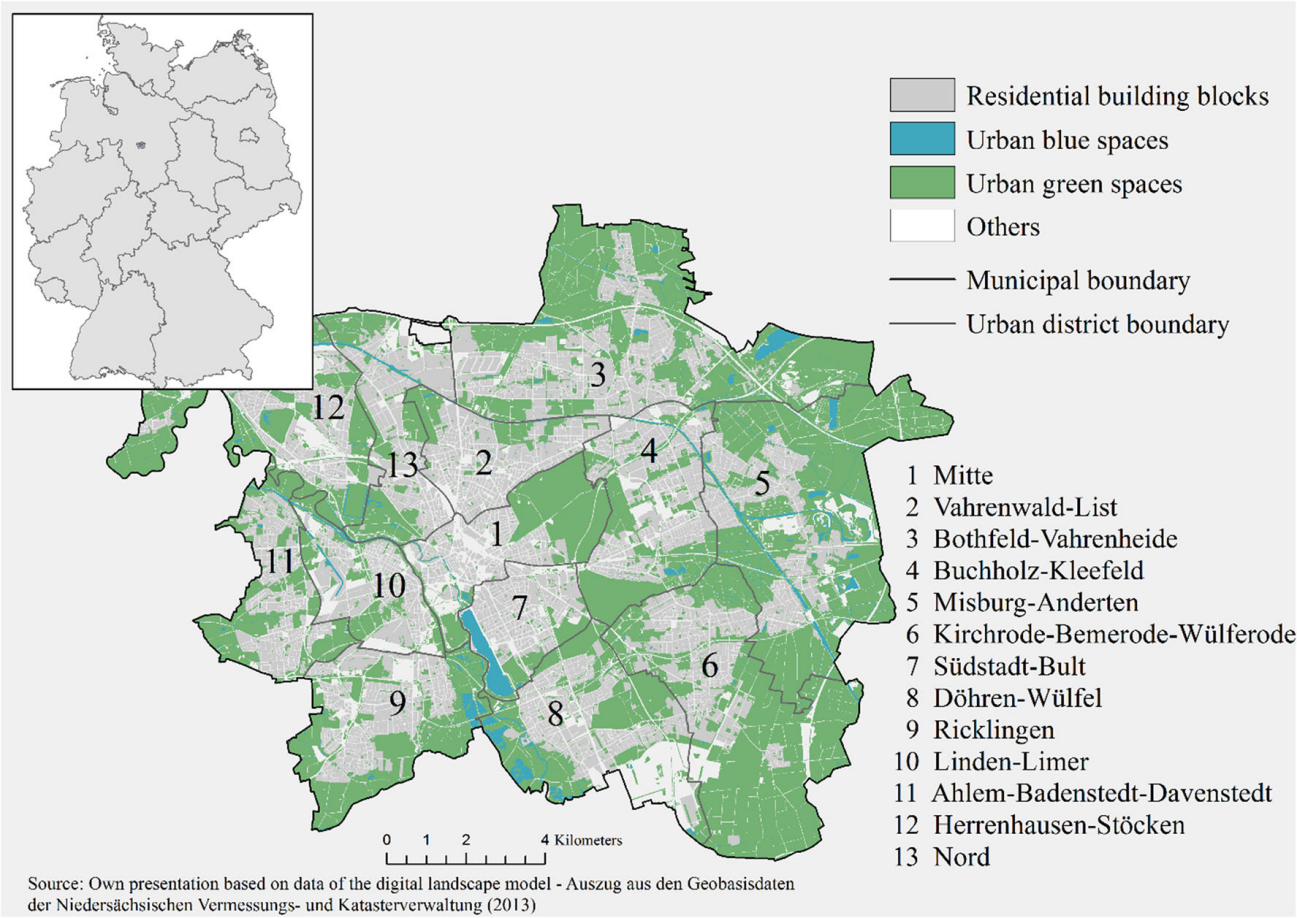
Relevant land cover in the study area and its urban districts

The city of Hannover, Germany, was selected for this case study because it provides a particularly diverse interplay of green and blue biotope types. The administrative area of the city extends over 204.2 km^2^, with about 60 km^2^ occupied for residential purposes. Areas of about 100 km^2^ and 6 km^2^ are covered by green vegetation and urban blue spaces, respectively (see Fig. 1). The city was populated by 541.773 inhabitants in 2018 (see Fig. S4; Statistikstelle der Landeshauptstadt Hannover 2018). Hannover is divided into 13 urban districts and 49 boroughs. Particularly, urban districts neighbouring the urban centre have dense building structures that correspond to the population density. Moreover, the city is traversed by networks of smaller and larger blue and green spaces, including the floodplain of the river Leine.

### Research design

**Fig. 2:**
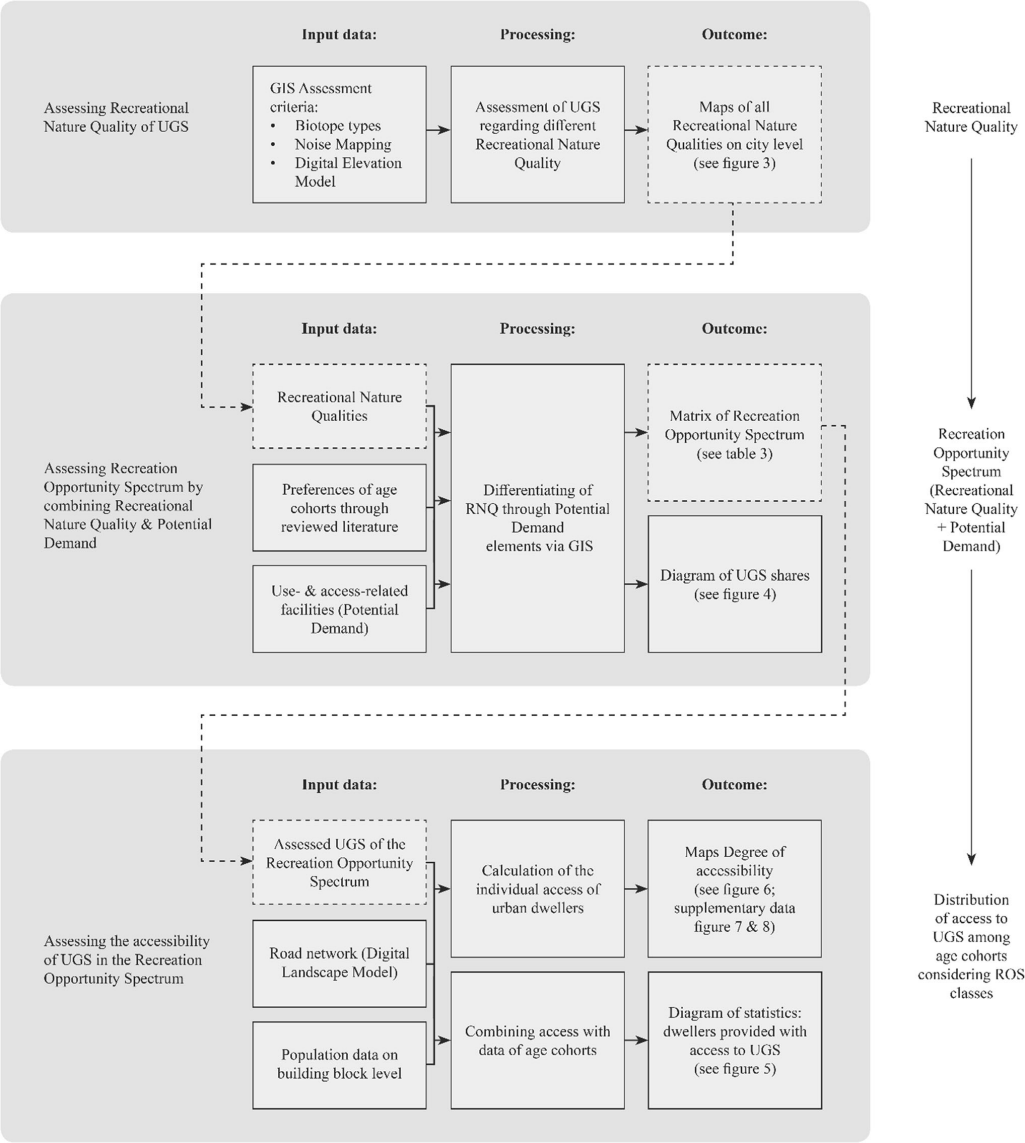
Research design

The research design combines three analyses, which were applied via ArcGIS to assess the distribution of access to UGS with a recreation opportunity spectrum (ROS) (Cortinovis and Geneletti 2018; Cortinovis et al. 2020) among residents at the building block level. This spectrum combines recreational supply through an analysis of RNQ and the availability of age-related infrastructure (PD)(see Figs. 1, 2, S3). First, the analysis of RNQ aims to evaluate all UGS for nine RNQs. The different qualities are defined by assessment criteria that are partly based on those proposed by van den Bosch et al. (2015). Second, ROS levels were evaluated by combining RNQs and Potential Demand. Potential Demand has been defined here as the changing age-related recreational use capacity and preferences of humans during their life to obtain recreation that reflects a user-related dimension. This dimension considers the demands of three age cohorts, following common practice in peer-reviewed publications.

Third, the network analysis assessed the distribution of individual access to UGS fulfilling relevant ROS classes which are composed of age-related Potential Demand and RNQs (see Fig. S5).

### Assessing the RNQ of UGS

The GIS-assessment of UGS classifies the natural inventory of Hannover using RNQ. Studies of RNQ categories and their relation to human well-being have revealed different degrees of importance for dwellers’ stress relief and health (Berggren-Bärring and Grahn 1995; Björk et al. 2008; Annerstedt et al. 2012; van den Bosch et al. 2015). RNQ, as defined by van den Bosch et al. (2015), used very low noise values (see Table 1) that are rarely found in major cities. Therefore, three additional RNQs^2^ were added to increase the range of RNQs (see Table 1) that might be perceived in urban settings. These new RNQs contain noise levels complying with the recommended thresholds for European regions by the WHO in the recently published Environmental Noise Guidelines (2018). In Germany, the Federal Immission Control Act determines certain noise values for human-dominated spaces, such as urban areas (63 dB) and general residential areas (55 dB) (Bundesministerium für Umwelt, Naturschutz und Reaktorsicherheit [BMUNR] 1998).

**Table 1:** Assessment criteria and description of RNQ based on van den Bosch et al. (2015)

Assessment criteria	Serene	Spacious	Wild	Soughing openness	Soughing wild	Soughing others	Lush	Culture	Common
Noise	< 40 dB^a^	< 40 dB^a^	< 40 dB^a^	< 55 dB^b^	< 55 dB^b^	< 55 dB^b^	–	–	–
Size	–	> 25 ha	–	> 25 ha	–	–	–	–	–
Slopes	–	> 10 degrees	> 10 degrees	–	> 10 degrees	–	–	–	–
Biotope types	Forests	Forests	Forests	Forests	Forests	–	Forests	–	–
	Heath and grassland	Heath and grassland	Heath and grassland	Heath and grassland	Heath and grassland	–	–	–	Heath and grassland
	Fen, Raised- and transitional bogs	Fen, Raised- and transitional bogs	–	Fen, Raised- and transitional bogs	–	–	Fen, Raised- and transitional bogs	–	–
	–	–	Inland waters	Inland waters	Inland waters	–	–	–	Inland waters
	–	–	Dry and wet ruderal meadows	–	Dry and wet ruderal meadows	–	–	–	Dry and wet ruderal meadows
	–	–	Hedges and groves	–	Hedges and groves	–	–	–	Hedges and groves
	–	–	–	–	–	Horticultural biotopes	–	Horticultural biotopes	–
	–	Bare rock	Bare rock	Bare rock	Bare rock	–	Bare rock	–	–
	–	–	–	–	–	Nature reserve	Nature reserve	Nature reserve	–
	–	Arable land (without horticulture)	–	Arable land (without horticulture)	–	–	–	Arable land (without horticulture)	–
	–	Beaches, dunes and sand plains^c^	–	Beaches, dunes and sand plains^c^	–	–	Beaches, dunes and sand plains^c^	–	–
Description of the perceived sensory dimensions of the RNQ	“A place of peace, silence, and care. Sounds of wind, water, birds, and insects. No rubbish, no weeds, no disturbing people”^d^	“A place offering a restful feeling of "entering another world", a coherent whole, like a beech forest”^d^	“A place of fascination with wild nature. Plants seem self-sown. Lichen and moss-grown rocks, old paths”^d^	A place of twittering birds and other sounds. Coherent nature offering vistas over open areas	A refuge hiding for looks while hearing wispering noises from everywhere	A place witnessing human existence in natural environment. Diverse sounds in wide-ranging green. Insects and birds.	“A place rich in species. A room offering a variety of wild species and animals and plants”^d^	“The essence of human culture. A historical offering fascination with the course of time”^d^	“Easily accessible and busy place offering good oppurtunities meeting people and watch one another”^d^

^a^Comprises immission values: < 45 dB road traffic, < 44 dB light rail, < 54 dB rail, < 40 dB aircraft, < 45 industry

^b^Comprises immission values: < 53 dB road traffic, < 54 dB light rail, < 54 dB rail, < 45 dB aircraft, < 55 industry

^c^These biotopes are not located in the study area

^d^Björk et al. (2008) p. 3; Annerstedt et al. (2012), p. 4; van den Bosch et al. (2015) p. 7979

The RNQ *Spacious* is derived from the perceived sensory dimension ‘Space’. This dimension has been defined as “spacious and free” and as an area where “one is not disturbed by too many roads and paths” (Grahn and Stigsdotter 2010, p. 268) based on empirical evidences of a geo-coded questionnaire. This described variable is, following Grahn and Stigsdotter (2010), the most important character with a high factor loading in the dimension ‘space’. A minimal size of’ > 25 ha’ as an additional assessment criteria has been defined for *Spacious* by a first follow-up investigation by Annerstedt et al. 2012 due to its loading. In line with the operationalisation by Annerstedt et al. (2012) the RNQ *Spacious, Wild* and *Soughing wild* has been defined by the assessment criteria ‘slopes > 10 degrees’. This is derived from the abovementioned study revealing that the perceived sensory dimension ‘Nature’ (*Wild*)^3^ is characterised by “hilly areas” (Grahn and Stigsdotter 2010, p. 267) and the dimension ‘Space’ is “sheltered from the wind” (ibid, p. 268) which has been interpreted as landscapes with slopes. In the same manner Annerstedt et al. (2012) operationalised results of the geo-coded questionnaire and translated them into GIS-assessment criteria.

The RNQ analysis dealt with different assessment criteria and evaluated the values of noise in the environment, size of each UGS, slopes of the surface, and biotope types (see Table 1). Different RNQs combine several features and may affect visitors or nearby residents in diverse ways. Other studies used the CORINE dataset, which has a resolution of 25 m x 25 m (1:100.000) (Annerstedt et al. 2012; van den Bosch et al. 2015). The feature-structured vector data (DLM) are independent of the scale and feature extent. Our data ensures a maximum deviation of 3 m and implies a higher position accuracy, which is significant for the subsequent analyses to minimise deviations in distance and service area characteristics.

### Assessing the potential demand of age groups

The Potential Demand depicts the interactional dimension of Environmental Justice such that “UGS should allow for all visitor groups—regardless of age and cultural background—to interact freely and safely” (Kabisch and Haase 2014, p. 137). Human needs and ability to reach for green spaces change throughout the life cycle. With advancing age, more emphasis is put on the perception of nature as a place for inspiration or to pursue physical activities (Plieninger et al. 2013). In our study, elderly people were defined as persons aged ≥ 65 years with primary residence in the study area, for comparability with other studies and considering life course changes after retirement (Arnberger et al. 2017). In contrast to all other age cohorts, research has already explored user behaviours and demands of elderly people related to UGS, including health-related specific needs (Table 2). While most studies suggest benches as one of the most important elements for elderly people (ibid., Joseph and Zimring 2007; Aspinall et al. 2010), there are no related data available at the responsible planning department of Hannover. Therefore, benches as a supporting infrastructure for elderly people cannot be regarded in this assessment.

**Table 2:** Assessment criteria of the potential demand

Potential demand	Children	Elderly people	Majority
Walking distance	500 m^a^	500 m^b^	835 m
Minimum size	0.5 ha	0.5 ha	0.5 ha
Maximum noise level	–	< 55 dB^c^	< 55 dB^c^
Special preference	-Playgrounds/sports ground^g^	-Shade and cool air providing places: forests, urban parks and blue spaces^d^	
		-Allotment gardens^e^	
		-Easily accessible path on UGS^f^	

^a^Stessens et al. (2017), p. 334; WHO (2016), p. 25

^b^Arnberger et al. (2017), p. 107; WHO (2016), p. 25

^c^Comprises immission values: < 53 dB road traffic, < 54 dB light rail, < 54 dB rail, < 45 dB aircraft, < 55 industry

Cerin et al. (2013), p. 86; Hung and Crompton (2006), p. 291

^d^Amberger et al. (2017), p. 102; Aspinall et al. (2010), p. 1025

^e^Leaver and Wiseman (2016); Milligan et al. (2004)

^f^Arnberger et al. (2017), p. 102; Joseph and Zimring (2007) p. 78; Kaczynski Johnson and Saelens (2010) p. 415; Aspinall et at. (2010), p. 1025

^g^Semenzato et al. (2017); Potwarka and Kaczynski (2008); p. 348, Oreskovic et al. (2015), p. 255

Further, considering age as a factor in the assessment of Potential Demand, children were defined as dwellers below 15 years^4^ due to their higher demand for nearby UGS and their lower mobility through higher dependence on a legal guardian (Maas et al. 2005; Duncan et al. 2011). The age group constellation is significantly influenced by pre-aggregated municipal data. Thus, the maximum proximity to UGS with a minimum size of 0.5 ha and a preference for playgrounds in green environments is defined by a walking distance of 500 m, based on the WHO recommendations (2010, 2016). Further, several studies have verified the positive effects between nearby UGS and children’s health (Potwarka and Kaczynski 2008; Semenzato et al. 2010; Markevych et al. 2014; Oreskovic et al. 2015). All studies revealed a significant correlation between the absence of UGS (≥ 0.5 ha) located in a range of 500 m/1000 m to children below 19 years and behavioural problems and higher systolic blood pressure level (ibid.; Younan et al. 2016). Amoly et al. (2015) found that longer play times in green spaces were linked to better interpersonal relationships among children of the same age, and also denoted a link between low access to residential UGS and poor mental health.

Dwellers aged between 15 and 64 years are classified as the ‘majority’. This three-pronged age group classification is based on the research literature dealing with age groups and UGS. Most of the research focus on children, elderly people or does not take age specifically into account. According to the WHO recommendations (2010, 2016), we set a Potential Demand baseline for ‘majority’ with a 0,5 ha as minimum size, a distance of 500 m (835 m walking distance) and noise limit in urban greenery. Hence, the next UGS should be reached in 10 min,^5^ which corresponds to a maximum walking distance of 835 m at a walking speed of km hr-5. Several cities have already recommended a maximum linear distance of 500 m to the nearest UGS (Grunewald et al. 2017; Richter et al. 2017). Linear distances generated by buffers are shorter than walking distances because of existing barriers and varying courses of roads in the urban landscape. Richter et al. (2016) revealed that a 300 m linear distance and 500 m walking distance in major cities in Germany correspond to a ratio of 1:1.66.

All UGS were merged with all age group-related assessment criteria of the Potential Demand shown in Table 2. Then the total UGS area for the specific age groups were calculated.

### Assessing the ROS

The assessment of green spaces merges information of each UGS area (≥ 0,5 ha) regarding its RNQ and Potential Demand to evaluate their ROS. Therefore, the ROS consists of the RNQ and Potential Demand. Based on the varying health and nature quality aspects of the analysed RNQ, an additional classification was integrated that encompassed three ROS classes (low/medium/high) for each age group. To reduce the number of different types of ROS, certain RNQs that show similarities were bundled to create an ROS class (see S3). Thus, 27 types of ROS were reduced into nine ROS classes. The ‘high ROS’ class shows supporting features that can increase the recreational potential; research results have indicated that the nature qualities *Serene*, *Spacious*, and *Wild* are integral for stress relief (Grahn et al. 2005; Grahn and Stigsdotter 2010; Annerstedt et al. 2012; van den Bosch et al. 2015). The class ‘medium ROS’ is characterised by a good quality of the UGS because it integrates the RNQ elements of *Soughing openness*, *Soughing wild*, and *Soughing others*. These qualities possess the same assessment criteria as mentioned above but have a higher noise limit, which in turn reduces its recreational potential. The class ‘low ROS’ contains all remaining qualities that have not shown any benefit for stress relief (see Table 1; Fig. S3). Based on the constellation of the ROS, each type was merged and intersected with the assessment criteria of relevant RNQ and Potential Demand. Afterwards the total UGS area for each ROS type was calculated.

### Assessing the accessibility of UGS

The network analysis aims to assess the access to UGS in walking distance based on the road network, population data and ROS (see Fig. S5). A new network data set was built with requirements such as motorways are excluded and no U-turns are allowed. In the next step, buffers were implemented around UGS to create catchment areas like two-lane streets. Following this, access points to UGS were defined with the catchment areas and the new network data set. In the main part of the network analysis services areas of UGS fulfilling ROS were created based on age group-related maximum walking distance and the aforementioned outcomes (see Fig. S5). The walking distance was measured from the middle of each building block to the nearest access point of the UGS and was linked to the population data.

## Results

### RNQ of UGS in Hannover

**Fig. 3:**
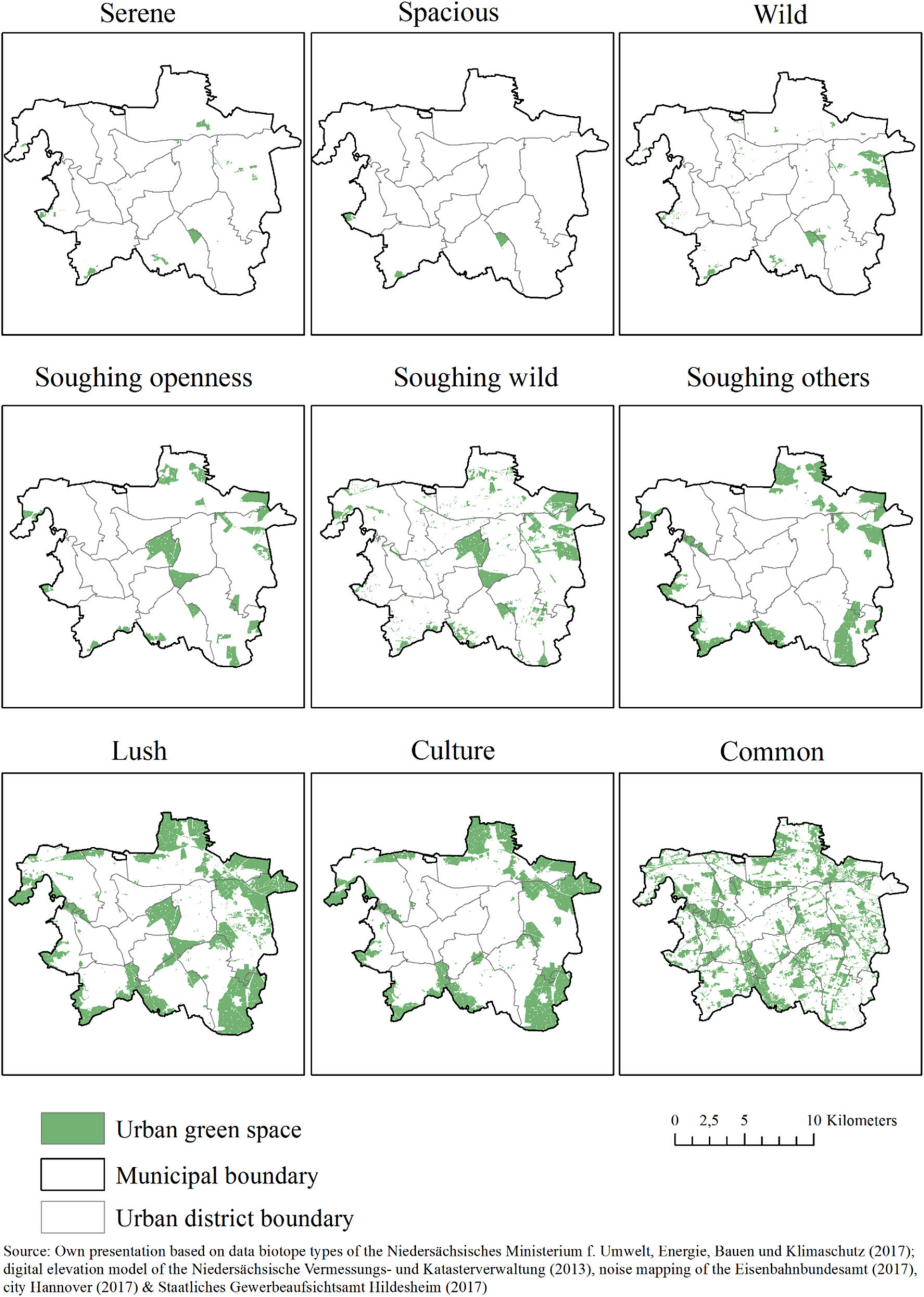
Maps of Hannover showing the distribution of nine RNQ

Results indicate that UGS in Hannover can be evaluated based on nine qualities. The total area of the RNQs vary considerably. The RNQs *Serene* (2.4%), *Spacious* (1.2%), and *Wild* (5.9%) characterise a rather low area share of the overall UGS and are located on the outskirts (see Fig. 3). This is linked to higher threshold values of the assessment criteria, as opposed to the other RNQs (see Table 1). In contrast, the RNQ *Lush* amounts to 57.8% of the highest share of Hannover’s overall UGS area.

The largest biotope patch assessed by RNQ covers 294 ha and is located in the east of Hannover. In contrast, the UGS assessed by *Common* shows few larger or medium-sized green spaces and evenly distributed, predominantly small green patches (see Fig. 3) due to a median size of 0.6 ha. UGS located at the periphery are predominantly assessed by *Soughing others* and *Culture*, which can be derived from the biotope type ‘Nature reserve’ that are commonly situated on the outskirts.

### Usable UGS for different age groups

The GIS-assessment indicates substantial differences between the shares of the total UGS area and the RNQ areas that can be used by different age cohorts.

**Fig. 4:**
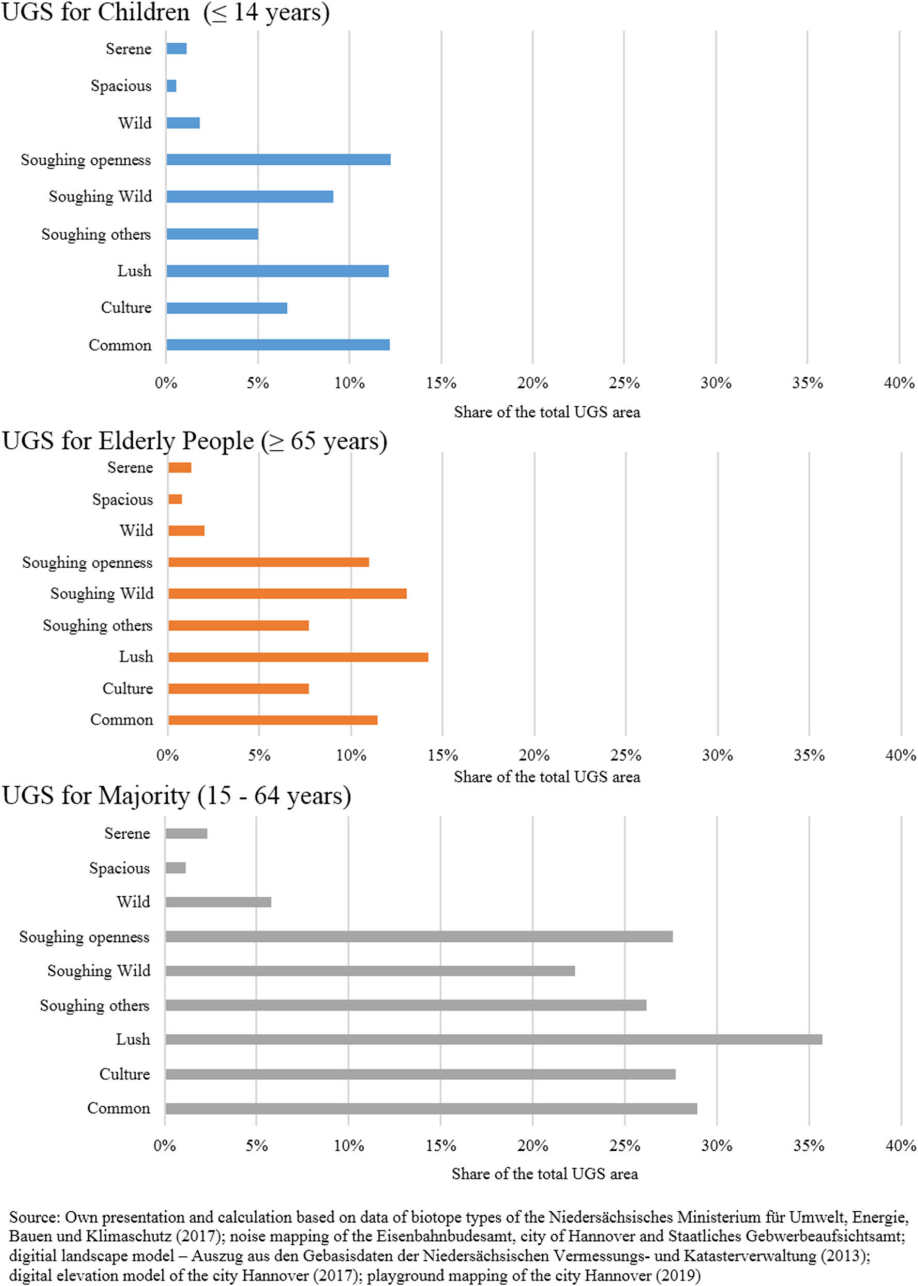
Share of the UGS fulfilling the recreation opportunity spectrum. Depicted green spaces represent intersections of nine RNQ and three Potential Demand types

UGS offer less features to fulfil the demands and preferences of children (23.3%) and elderly people (24.0%) in Hannover, while the ‘majority’ (57.7%) are provided a rich variety. In the case of middle-aged dwellers, six out of nine types of ROS represent 25% of the overall UGS area. UGS for children in each ROS type are underrepresented in contrast to green spaces fulfilling the requirements of elderly people, with the exception of the *Common* and *Soughing openness* types (see Table 3 and Fig. 4).

**Table 3:** Matrix of the recreation opportunity spectrum (ROS). Depicted green spaces represent intersections of nine RNQ and three Potential Demand types

	Potential Demand					
	Children (≤ 14 years)		Elderly People (≥ 65 years)		Majority (15 – 64 years)	
Age groups	Share of overall UGS	Area size	Share of overall UGS	Area size	Share of overall UGS	Area size
Children (≤ 14 years)	2414.6 ha	23.3%	–	–	–	–
Elderly People (≥ 65 years)	–	–	2490.8 ha	24.0%	–	–
Majority (15 – 64 years)	–	–	–	–	5989.1 ha	57.7%
**Recreational nature qualities**						
Serene	119.1 ha	1.1 %	134.7 ha	1.3 %	242.5 ha	2.3 %
Spacious	59.7 ha	0.6 %	78.7 ha	0.8 %	119.7 ha	1.2 %
Wild	193.3 ha	1.9 %	210.0 ha	2.0 %	599.9 ha	5.8 %
Soughing openness	1272.1 ha	12.3 %	1142.4 ha	11.0 %	2866.0 ha	27.6 %
Soughing Wild	947.3 ha	9.1 %	1352.0 ha	13.0 %	2315.9 ha	22.3 %
Soughing others	521.3 ha	5.0 %	797.5 ha	7.7 %	2717.0 ha	26.2 %
Lush	1262.6 ha	12.2 %	1478.0 ha	14.2 %	3709.2 ha	35.7 %
Culture	685.1 ha	6.6 %	799.4 ha	7.7 %	2882.2 ha	27.8 %
Common	1268.7 ha	12.2 %	1190.5 ha	11.5 %	3002.2 ha	28.9 %

Source: Own calculation and design based on following data sets:

-Noise mapping of City Hannover (2017), Eisenbahnbundesamt (2017) and Staatliche Gewerbeaufsichtsamt (2017)

-Digital elevation model of the city Hannover (2017)

-Digital landscape model of Niedersächsische Vermessungs- and Katasterverwaltung (2013)

-Playground mapping of the city Hannover (2019)

-Biotope mapping of Niedersächsisches Ministerium für Umwelt, Energie, Bauen and Klimaschutz (2017)

-Municipal demographic statistics of Statistikstelle der Landeshauptstadt Hannover (2018)

Remark: The area sizes of the individual Potential Demand shown in the matrix must be independently considered because the RNQ of UGS share similarities such as spatial intersections. Hence, these cannot be accumulated to obtain the total area for example

Furthermore, Fig. 4 indicates a similar pattern of the distribution of the ROS types regarding the specific age groups and taking into account the aforementioned different overall shares of the UGS area per age group.

In general, types with a higher ROS such as *Serene*, *Spacious*, and *Wild* are at least represented across age cohorts, compared to the remaining types.

### Distribution of access to UGS among dwellers in Hannover

**Fig. 5:**
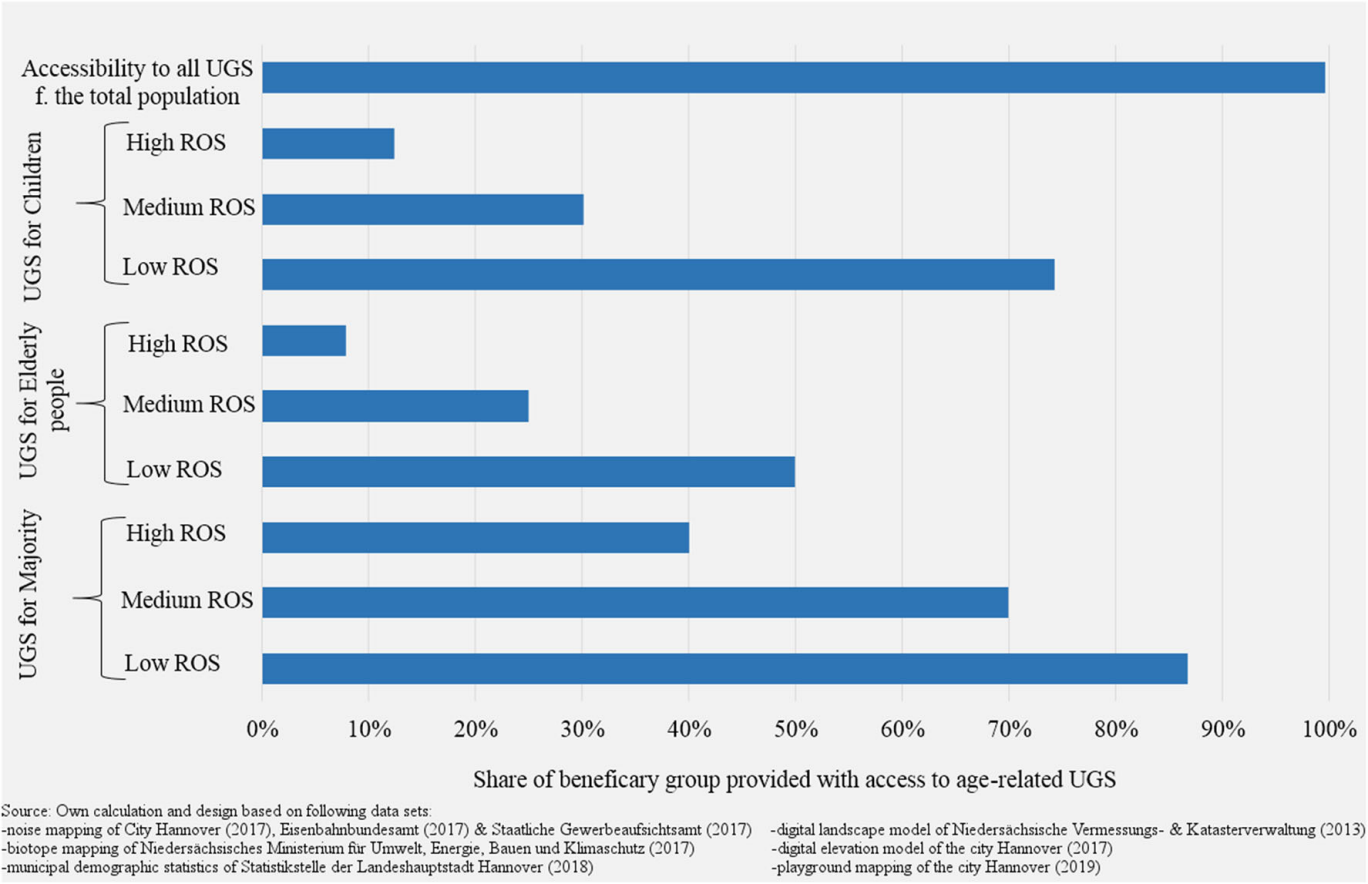
Distribution of access to UGS on city level regarding age groups and different classes of the recreation opportunity spectrum (2018)

Almost every citizen has access to UGS at a walking distance of 835 m in Hannover. Only 0.4% of the total population lives out of the service area (Fig. 5).

Available access to UGS varies considerably between age-related ROS and for single classes. Accordingly, the shares of beneficiary groups with access to age-related UGS decrease while to ROS increases.

**Fig. 6:**
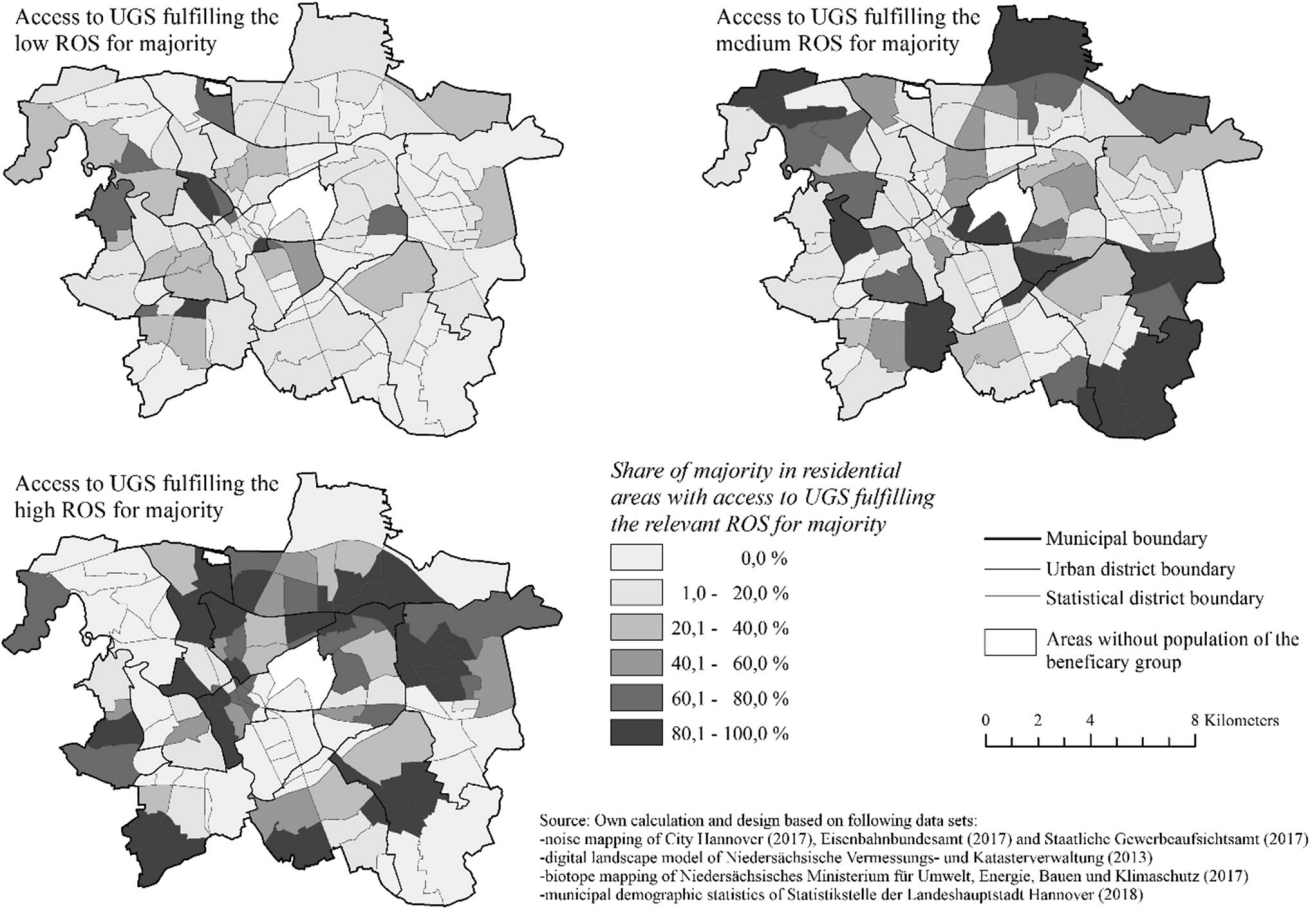
Statistical districts with access to UGS fulfilling the relevant recreation opportunity spectrum (ROS) for majority (2018)

The maps in Fig. 6 show the indicated data from Fig. 5 (similar to Figs. S1 and S2). The figure below shows the spatial distribution of the proportions of the ‘majority’ who have access to UGS fulfilling a certain ROS. All values were measured at the building block level, but for practical reasons, they are shown at the statistical district level. Our analysis indicated that overall low (0.0–20.0%) access to UGS (but notwithstanding the ROS) were found in large parts in the south of the inner city, the eastern and western periphery.

The distribution of access to UGS with a high ROS for the ‘majority’ is characterised by several large spatial concentrations of statistical districts that notably result in an agglomeration of higher proportions in the northern and northeast regions of Hannover. In addition, statistical districts with 60.1–100.0% are located especially on the outskirts, in the western part of the inner city and in the north of Hannover. These areas benefit from UGS including the forests of south-eastern part and several large allotment garden areas along the Mittelland Canal.

Access to UGS with a high ROS is characterised by an unequal spatial distribution among all considered age cohorts; this is due to an insufficient provision of access to UGS, and an absence of accessible green spaces with minimal quality as well as the requirements for recreation of the aforementioned urban areas. The assessment indicates a fragmented, citywide low provision of access to UGS for all ROS classes comprising suitable features for children. The distribution of access to UGS with high ROS for children is represented by a few spatial concentrations. Accordingly, only two out of 119 statistical districts (eastern and south-eastern city) are provided with 80.1–100.0% provision of access to green spaces with a high ROS (see Fig. S1).

The assessment shows an inequitable distribution and deficient provision of accessibility among elderly people in Hannover (see Fig. S2). In particular, the northeast of Hannover and areas around the urban forest Eilenriede next to the inner city are characterised by relatively coherent areas with a moderate proportion of elderly people who have access to UGS with a medium ROS. Notably, only the few statistical districts in the south east show a share of 83% of elderly people who can access UGS with a high ROS.

## Discussion

This study assessed UGS in Hannover by examining their RNQ and Potential Demand and the distribution of access to these evaluated spaces. The study explored the distributive and interactional dimensions which bolster a more equity-oriented and embodied perspective of Environmental Justice. Furthermore, the RNQ approach allowed the assessment of UGS according to predefined criteria that led to individual RNQs offering different benefits for human health. Based on the consideration of age-related demands and preferences, UGS could be assessed by their physical infrastructure and conditions, which allows us to draw conclusions regarding the interactional dimension of Environmental Justice in terms of safety and capabilities for users in the respective space. In the following we discuss our results in front of the three research questions. Afterwards we reflect our procedure and discuss methodical implications.

Our results concerning the spatial distribution of UGS and its RNQ (research question 1) illustrated that blue and green spaces in Hannover could be evaluated based on nine different RNQs. The most common nature quality is *Lush* (57.8%), offering high biodiversity, especially in forests, nature reserves, and bogs on the periphery. However, these UGS are rather consistently located in the central urban forest Eilenriede and on the outskirts. Furthermore, RNQs such as *Common* and *Culture* cover vast parts of the UGS. Björk et al. revealed that *Culture* and *Lush* were the most dominant nature qualities, detected to be in close proximity of 300 m to residents in suburban and rural Scania in southern Sweden (Björk et al. 2008; van den Bosch et al. 2015). In contrast, *Serene*, *Spacious*, and *Wild* can rarely be found in Hannover and Scania (ibid.). Björk et al. (2008) reported that *Lush*, *Serene*, *Spacious*, and *Wild* had significant effects on stress relief for residents living in proximity of 300 m, based on results from an extensive public health survey conducted in 2004. Subsequent longitudinal studies have proved the positive effect of *Serene* and *Spacious,* attributable to the increased physical activity of residents (Annerstedt et al. 2012; van den Bosch et al. 2015). The risk-reducing effect of *Serene* on mental health disorders was only statistically significant for women (ibid.). Additionally, Grahn et al. (2005) found that *Serene*, *Spacious*, and *Wild* are the most important RNQs for stress relief. In relation to these findings on health statistics, our assessment of RNQs indicates that only a low percentage of UGS (*Serene*, *Spacious*, *Wild*) in Hannover show statistical benefits on stress relief, mental health by promoting increased physical activity among nearby residents. The integrated perceived sensory dimensions of RNQ that are used in several Swedish cities and in Baoji, China (Berggren-Bärring and Grahn 1995; Grahn et al. 2005; Grahn and Stigsdotter 2010; Gao et al. 2019) were also found in Hannover (see Fig. 3).

Regarding our second research question, the multi-criteria analysis considering demands and preferences indicated significant gaps between the share of UGS that provide suitable opportunities for age-related recreation. In total, 23.3% and 24.0% of the overall green spaces provide suitable recreational green areas for children (0 – 14 years) and elderly people (≥ 65 years), respectively. Meanwhile, dwellers aged between 15 and 64 are provided with more than double the share of UGS (57.7%), fulfilling the specific Potential Demand at the city level. Our GIS-assessment indicates differences, and some discrimination, in the availability of UGS suitable for (age-related) recreation.

The GIS network analysis concerning the accessibility of UGS relating to different age groups (research question 3) revealed that in Hannover, 96.6% of residents could access an UGS regardless of its size or quality, at a walking distance of 835 m. While several studies have assessed the accessibility of UGS based on linear distance (Herzele and Wiederman 2003; Krekel et al. 2016; Grunewald et al. 2017; Wüstemann et al. 2017; Cortinovis et al. 2020), our analysis calculated the access to the nearest UGS by the network analysis, which also considered barriers such as non-walkable streets. In relation to a prior study, 500 m in linear distance corresponds to 835 m in walking distance (Richter et al. 2017).

Wüstemann et al. (2017) found that 92.8% of the population lives at 500 m distances (linear distances) to UGS in major German cities, suggesting an above-average UGS accessibility for Hannover. However, in view of this study’s focus, we propose that elderly people and children have considerably less access to suitable UGS in Hannover in the sense of equity. With reference to health risks for these vulnerable population groups, there is an urgent need to improve the RNQ and Potential Demand of UGS in poorly serviced urban areas to increase environmental equity.

Children are provided at least by UGS area (23.3%) in Hannover that possess suitable features for them (see Table 3). However, the UGS per capita amounts to 344 m^2^/head for this age cohort, which is significantly higher than that for other age groups. Hence, this comparison indicates that children (0–14 years) live closer to suitable UGS than elderly people (24.0% of the overall UGS). However, UGS for vulnerable groups with a high ROS remain rare. In contrast, the share of dwellers with access to UGS with at least low ROS (86.8%) at an age-appropriate distance corresponds closely to the aforementioned citywide average.

These findings illustrate that UGS have to be critically considered. The mere existence of green areas in close proximity does not imply that every nearby living dweller can reach, use and enjoy this space. As we have shown green space features such as playground, walking distances, safe paths, biodiversity or reduced noise levels play a crucial role for use and access to UGS.

Notably, with respect to particular spaces, allotment gardens were identified as semi-public islands with high Potential Demand and RNQ values for all residents. This insight is a central aspect in the contemporary debate on densification and housing shortage.

One major drawback of our GIS-approach is that we were limited to assessing the accessibility of suitable UGS for different age groups and were unable to verify epidemiological outcomes for the suburban context data (De Jong et al. 2011; Annerstedt et al. 2012; van den Bosch et al. 2015). Another problem with this approach is that other possible GIS-assessment criteria are pertinent to recreational activities of elderly people but do not comply with all dwellers across age cohorts or other criteria. For example, following criteria have previously been proposed, including restaurants and toilets or avoiding dog-walking fields. While numerous studies have explored the nature-based recreations of elderly people (Milligan et al. 2004; Hung and Crompton 2006; Kaczynski et al. 2010; Cerin et al. 2013; Leaver and Wiseman 2016; Arnberger et al. 2017; Aspinall et al. 2010) only a few have focused on the preferences and demands of children (Potwarka and Kaczynski 2008; Oreskovic et al. 2015; Semenzato et al. 2017). Research on perceived sensory dimensions and RNQ could be more enriched by collecting primary data through questionnaires via public participation geoinformation system or local questionnaires containing geo-codes. These possibilities may have the potential to proof and to develop this approach by catching the participants’ knowledge and relation to the explored space.

Difficulties arose because only a limited selection of sociodemographic indicators was available on building block level. To support Environmental Justice research more specified data on small-scale are needed such as disposable income, education level, recipients of state social benefits or unemployment.^6^ Another unexplored challenge is the potential conflict in UGS among different recreational activities or user groups: for example, leisure noises and activities caused by children playing and users seeking quietness in the same or neighbouring urban park (Wright Wendel et al. 2012). Nevertheless, the recommended and used assessment criteria show that children and elderly people are vulnerable groups, warranting focus in planning. In general and a positive signal is that, all data sets which have been integrated into this GIS-assessment are available in each municipality of the European Union due to several directives concerning data acquisition, such as the Environmental Noise Directive (The Council of the European Communities [CEC] 2002).

## Conclusions

This research explored the patterns of access to UGS qualities among different groups of residents in Hannover according to selected sociodemographic characteristics (representing distributional and interactional Environmental Justice). Results indicated that only a low share of green spaces were characterised by important RNQs offering settings that promote recreation among dwellers living in proximity to the UGS according to Annerstedt et al. 2012 and van den Bosch et al. 2015. Furthermore, children and elderly people were found to be disadvantaged in the provision of suitable UGS, with insufficient green space access for the latter. A key finding is, when we look at green spaces through a normative Environmental Justice lens that living in close proximity to an UGS does not implicate good access to UGS per se.

Such research approaches and findings could be integrated into informal urban planning instruments such as sectoral urban development plans and strategic concepts of open space and housing in the context of integrated urban development. Future research is needed to explore the relationship between RNQs and health benefits for different age cohorts, particularly for children. Prospective research and planning for UGS should represent nature-based solutions, ensuring fair accessibility for all potential users and providing equal opportunities for their recreation.

## Supplementary Information

Below is the link to the electronic supplementary material.

Supplementary file1 (PDF 963 kb)

## Footnotes

1 Minimum distance to an UGS equal or larger than 0,5 ha: Brunswick, 150 m; Freiburg and Munich, 300–500 m; Berlin, 500 m (DLR 2006, p. 16).

2 The RNQ *Soughing wild, Soughing openness and Soughing others* have been added due to large restricting impacts of the assessment criteria noise.

3 Annerstedt et al. (2012) translated the perceived sensory dimension ‘Nature’ into the RNQ *Wild. Soughing wild* is derived from *Wild* and reflects this quality in a reduced manner.

4 Other studies concerning children’s interaction with UGS used varying age group constellations: Markevych et al. (2014) (only 10 years), Amoly et al. (2015) (7–10 years), Dadvand et al. 2015 (7–10 years), Oreskovic et al. 2015 (11–14 years), Younan et al. (2016) (9–18 years), Stessens et al. (2017) (0–12 years).

5 The German council on landscape conservation sets up benchmarks for residence-related green spaces such as a minimum size of 0.5 ha, minimum proximity 10–20 min from residence and should provide 7 m^2^ per inhabitant. Furthermore the UGS should offer a variety for recreational opportunities from calm relation to active physical sports (DLR, 2006, p. 21).

6 An additional analysis of the sociodemographic data revealed no significant differences between the provision of access to UGS for dwellers with and without migrant descent among all ages on both city and building block levels. Migrant descent was originally used as a proxy for discrimination and displacement in the real estate market, possibly expressed by a certain segregated residential pattern (Comber et al. 2008).
