# Supplementary material for: Access to urban green spaces in Hannover: An exploration considering age groups, recreational nature qualities and potential demand
Source: Ambio. 2022 Dec 12;52(3):631–46. doi: 10.1007/s13280-022-01808-x (PMC9849547; doi:10.1007/s13280-022-01808-x)
Supplement: Supplementary file 1 — Supplementary file1 (PDF 963 kb) [file 13280_2022_1808_MOESM1_ESM.pdf]

Ambio

Supplementary Information

This supplementary information has not been peer reviewed.

Title: Access to urban green spaces in Hannover: An exploration considering age groups, recreational nature qualities and potential demand.

Authors: Raphael Weber, Annegret Haase, Christian Albert

Appendix

Figure S1: Statistical districts with access to UGS fulfilling the relevant recreation opportunity spectrum (ROS) for children (2018)
Figure S2: Statistical districts with access to UGS fulfilling the relevant recreation opportunity spectrum (ROS) for elderly people (2018)
Figure S3: Flowchart
Figure S4: Number of inhabitants per age group in statistical districts (2018)
Figure S5: Network analysis procedure

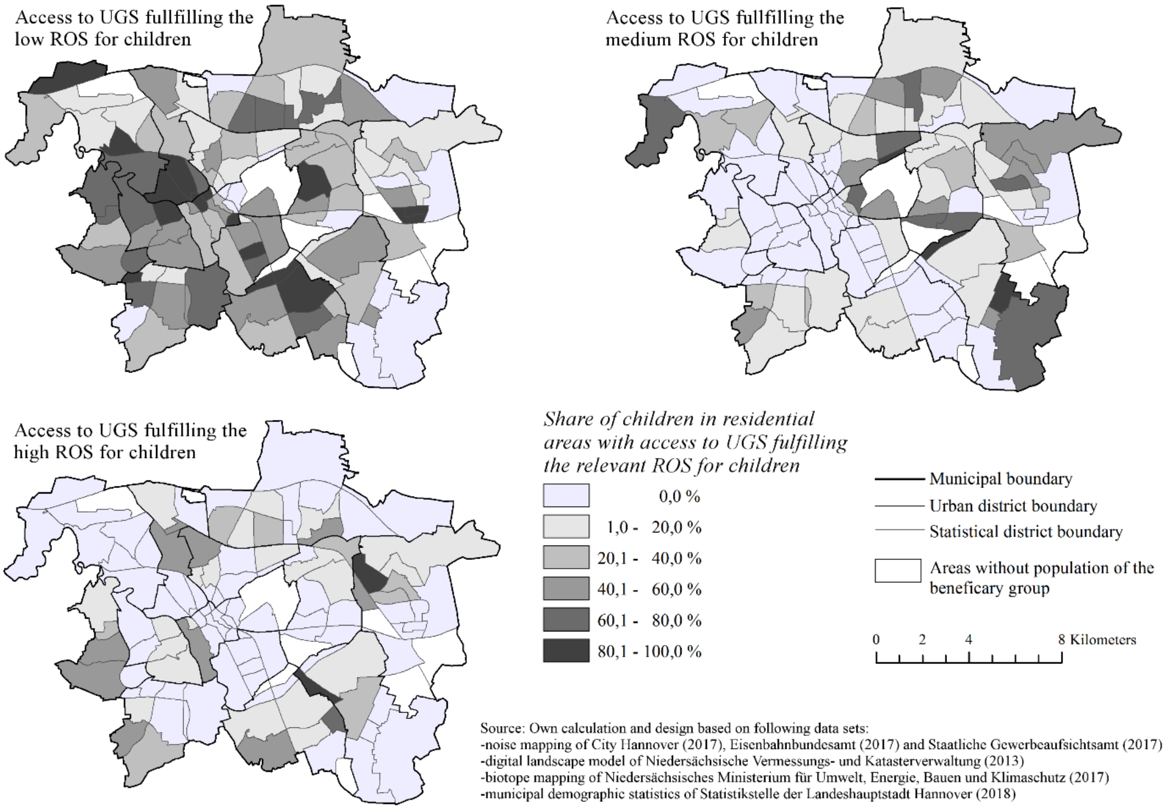

Figure S1: Statistical districts with access to UGS fulfilling the relevant recreation opportunity spectrum (ROS) for children (2018)

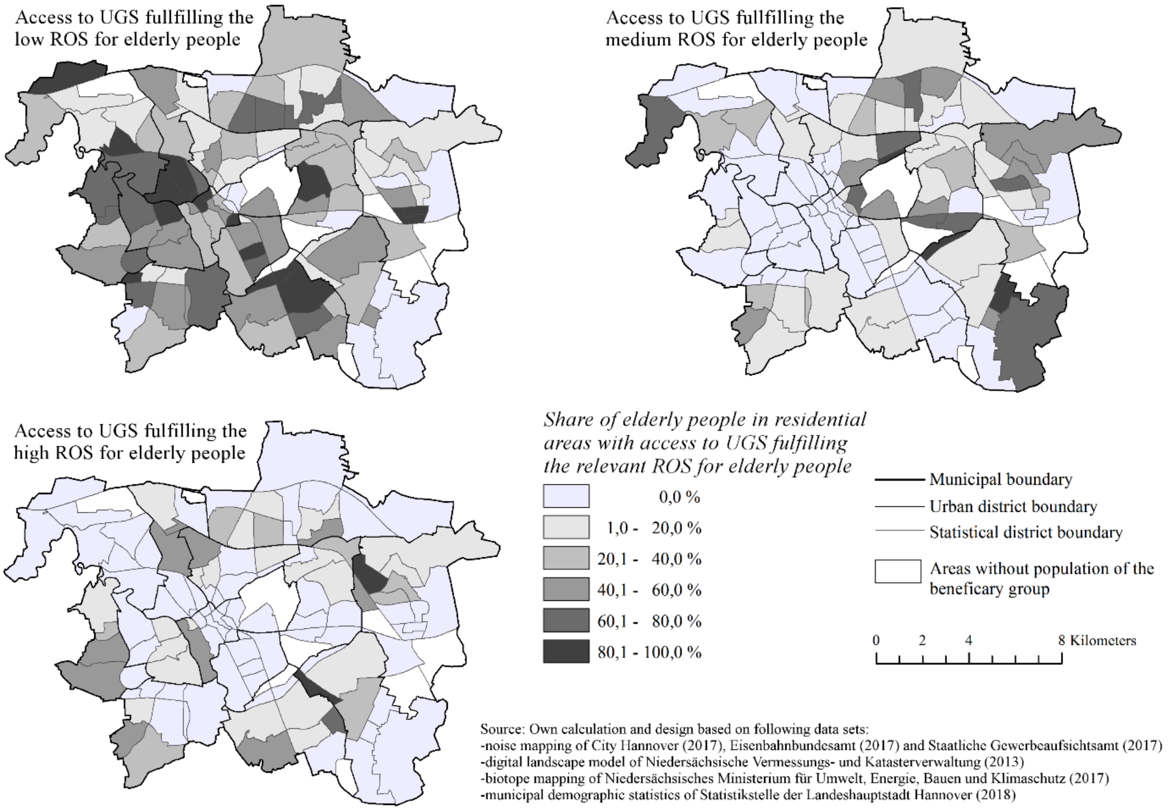

Figure S2: Statistical districts with access to UGS fulfilling the relevant recreation opportunity spectrum (ROS) for elderly people (2018)

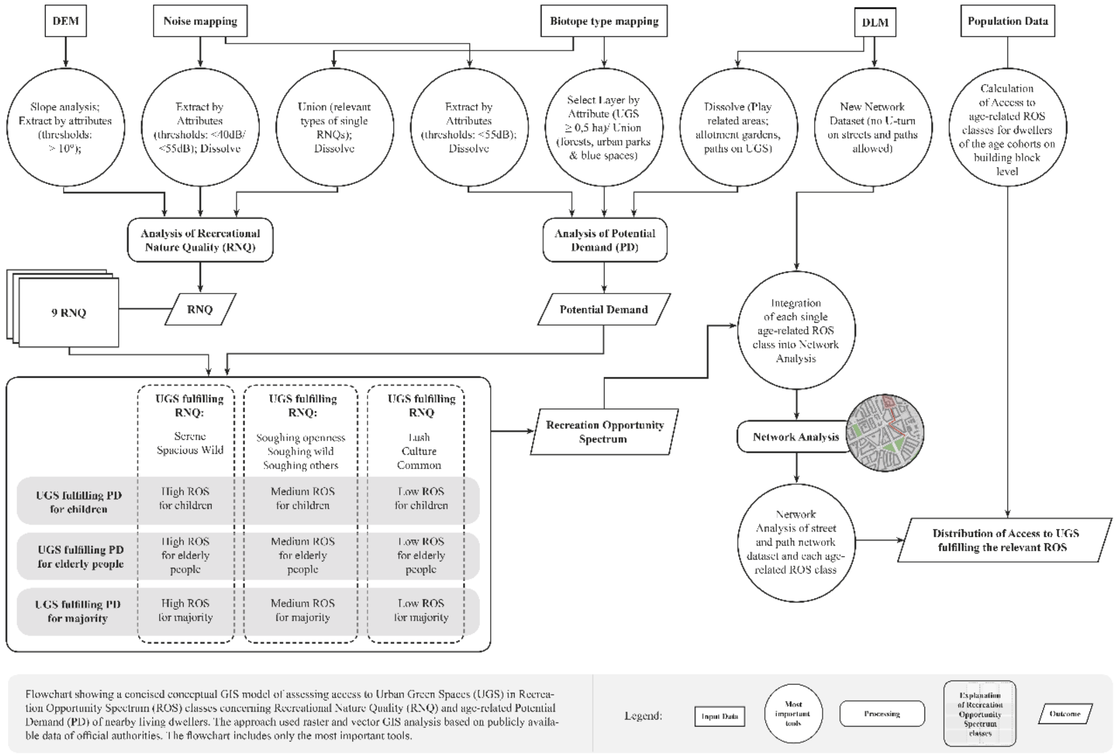

Figure S3: Flowchart

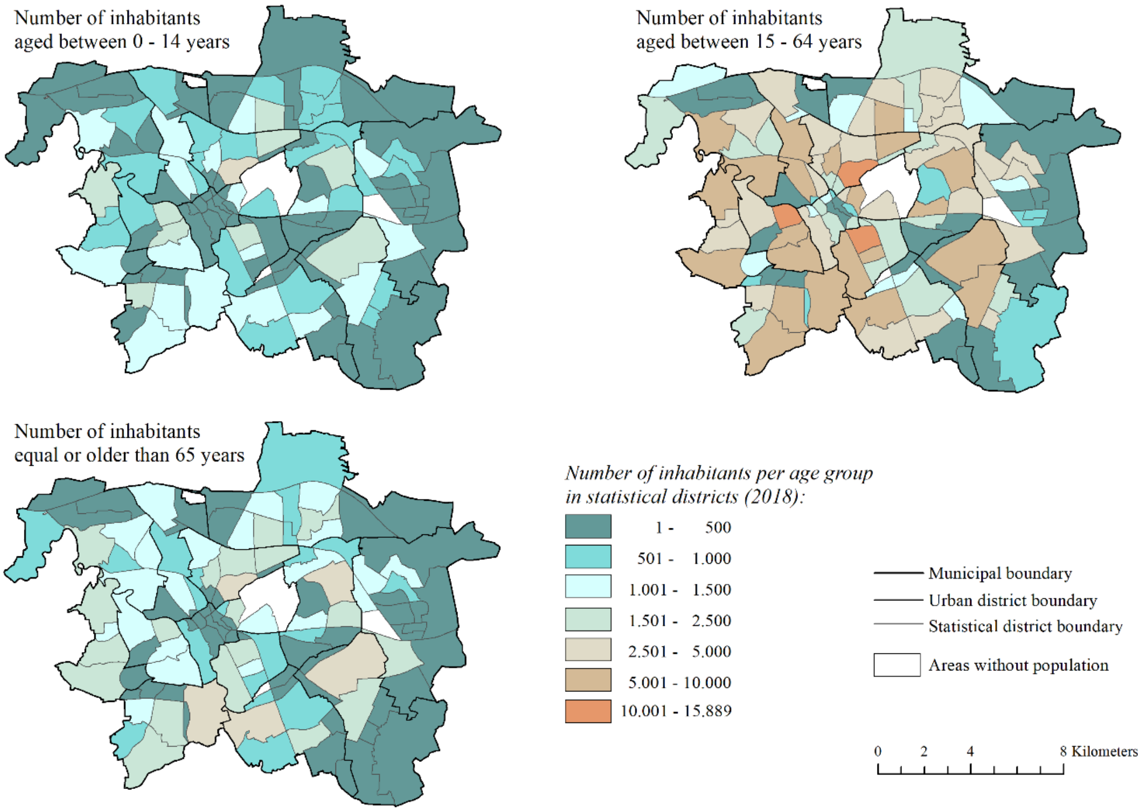

Source: Own calculation and design based on data sets of Statistikstelle der Landeshauptstadt Hannover (2018)

Figure S4: Number of inhabitants per age group in statistical districts (2018)

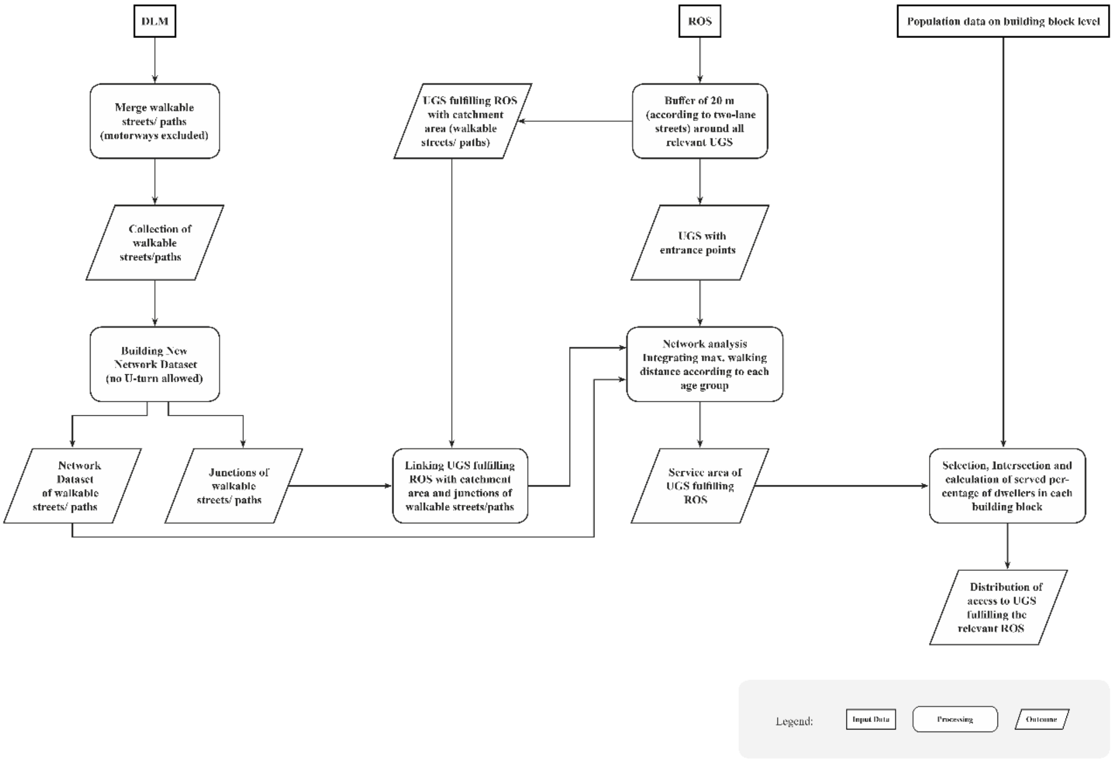

Figure S5: Network analysis procedure
